# Allosteric Inter-Domain Contacts in Bacterial Hsp70 Are Located in Regions That Avoid Insertion and Deletion Events

**DOI:** 10.3390/ijms23052788

**Published:** 2022-03-03

**Authors:** Michal Gala, Peter Pristaš, Gabriel Žoldák

**Affiliations:** 1 Department of Biophysics, Faculty of Science, P. J. Šafárik University in Košice, Jesenna 5, 04001 Kosice, Slovakia; michal.gala@student.upjs.sk; 2 Institute of Biology and Ecology, P. J. Šafárik University in Košice, Srobarova 2, 04154 Kosice, Slovakia; peter.pristas@upjs.sk; 3 Center for Interdisciplinary Biosciences, Technology and Innovation Park, P. J. Šafárik University in Košice, Trieda SNP 1, 04011 Kosice, Slovakia

**Keywords:** Hsp70, evolution, indels, conformational change

## Abstract

Heat shock proteins 70 (Hsp70) are chaperones consisting of a nucleotide-binding domain (NBD) and a substrate-binding domain (SBD), the latter of which binds protein clients. After ATP binds to the NBD, the SBD α/β subdomains’ shared interface opens, and the open SBD docks to the NBD. Such allosteric effects are stabilized by the newly formed NBD-SBD interdomain contacts. In this paper, we examined how such an opening and formation of subdomain interfaces is affected during the evolution of Hsp70. In particular, insertion and deletion events (indels) can be highly disruptive for the mechanical events since such changes introduce a collective shift in the pairing interactions at communicating interfaces. Based on a multiple sequence alignment analysis of data collected from Swiss-Prot/UniProt database, we find several indel-free regions (IFR) in Hsp70. The two largest IFRs are located in interdomain regions that participate in allosteric structural changes. We speculate that the reason why the indels have a lower likelihood of occurrence in these regions is that indel events in these regions cause dysfunction in the protein due to perturbations of the mechanical balance. Thus, the development of functional allosteric machines requires including in the rational design a concept of the balance between structural elements.

## 1. Introduction

In bacteria, heat-shock protein 70 (Hsp70, DnaK) is the essential, cell-survival response to a heat shock [1]. For the cellular function, DnaK acts as an ATP-regulated chaperone in cooperation with a co-chaperone DnaJ and a nucleotide exchange factor GrpE [2]. Both DnaJ and GrpE regulate limiting steps in the Hsp70 ATP cycle, while DnaJ accelerates ATP hydrolysis, while GrpE speeds up the replacement of ADP by ATP. Hsp70 is a key component of a protein quality network functioning as a molecular chaperone, i.e., it binds protein clients among other ways by recognizing motifs of five to seven amino acids. These motifs consist of hydrophobic amino acids flanked by the positively charged side chains [3]. 

The three-dimensional structure of bacterial Hsp70 mirrors its function; it is comprised of two folded domains—an N-terminal nucleotide-binding domain (NBD) and a protein-substrate binding domain (SBD) [4,5]. Hsp70 contains an unstructured C-terminal tail with lengths of high variability. A potential function of this unstructured tail has recently emerged in biochemical studies [6,7,8]. For the essential heat shock response of Hsp70, the allostery between protein substrate binding and ATP hydrolysis is vital. The allostery consists of coupling ATP/ADP states with substrate binding/unbinding dynamics. Namely, in the ATP form, protein substrates bind/unbind very quickly with a low affinity to the open binding site of the SBD. After ATP hydrolysis, helix B of the SBD closes the binding cleft and dramatically slows down the substrate binding/unbinding kinetics. For example, a model peptide substrate σ32-Q132–Q144-C-IAANS dissociates more than three orders of a magnitude slower in the ADP form than in ATP form [9]. In the closed form of DnaK, the substrate affinity increases predominantly due to this dramatic drop in the dissociation rate. Under certain nonequilibrium circumstances, an ultra-affinity between Hsp70 and a substrate can be achieved [10,11]. A non-allosteric Hsp70 variant with folded individually functional domains containing short inserts cannot rescue the temperature-sensitive phenotype of *ΔdnaK* knockout [12]. Hence, for the biological function of Hsp70, allosteric regulation of the capture and release of protein substrates is essential. Such need for domain allostery may also provide additional constraints in the evolution of the domains, i.e., the existence of co-evolution couplings between sites and correlated compensating mutations.

Hsp70s are found in all domains of life [1]; they are evolutionarily conserved—*E. coli* Hsp70 has 47.6% similarity with human Hsp70 1A isoform, 48.3% similarity with yeast Ssa and 51% to Hsp70 of archaeal bacterium *Aquifex aeolicus*. A high similarity between human and bacterial homologs indicates significant constraints to evolutionary diversification that limit sequence variability and diversity. The majority of previous studies on the Hsp70 evolution have focused on the phylogenetic relationships, evolution and co-evolutionary couplings in Hsp70 domains [1,13,14,15,16,17,18]. Smock et al. [7] used a statistical coupling analysis, SCA, to evaluate co-evolution between protein residues and functional divergence between sequences in protein sub-families. Employing this theoretical analysis, they discovered a group of co-evolving residues, which can be viewed as a feature of allosteric Hsp70. In line with an SCA-driven experimental approach, they found that these residues connect the functional sites of the two domains across a specific inter-domain interface. In other work, the Gierasch group [19] demonstrated that the allosteric landscapes of Hsp70s are shaped by evolutionary tuning of key interfaces and the tunability of Hsp70 functions by modulating allosteric interfaces through evolutionary diversification. Singh and Gupta [12] analyzed the role of naturally occurring inserts in Hsp70 of Gram-negative bacteria using complementation assays with temperature-sensitive (T(s)) mutants of CG800 (C600 dnaK103 + thr::Tn*10*) *E. coli* [20]. Their results indicate that the deletion or significant changes in these inserts completely eliminated the complementation ability of Hsp70 variants. Hence, these inserts are essential for the growth of *E. coli*. In addition, they analyzed a two amino acid insert found in some other bacteria but not in *E. coli*. The insertion of these two amino acids into the *E. coli* DnaK also led to its deactivation, which points to a high sensitivity of the Hsp70 function regarding insertions and deletions (indels). 

In our work, we focus on analyzing and explaining the pattern of indels in bacterial Hsp70. Given their probability and phylogenetic origin, we ask whether indel events are randomly distributed within Hsp70 domains. These events, per se, can be highly disruptive even for single-domain proteins; in a protein that relies on an allosteric coupling between two domains, these events can be even more severe. During the Hsp70 allosteric conformational changes, switching a part of the structure (e.g., helix B) from one interactive interface (binding cleft of the SBD) to a second interactive interface (lobe I of the NBD), requires two different pairing interactions. An indel event can be potentially highly disruptive for both interfaces due to collective shifts of the pairing interactions between both interfaces. 

Hence, a strong negative bias for indel events can be expected but had not been detected in previous studies. To this end, we performed multiple sequence alignments, analyzed indel locations, reconstructed ancestral protein sequences, and examined the correlation between interdomain contact residues involved in the allosteric transition and the existence of large indel-free regions in Hsp70. We found that evolutionary diversification by indels is significantly limited by the allosteric inter-domain interfaces due to the necessity of properly paired contact residues.

## 2. Results

### 2.1. Structural and Evolutionary Characterization of DnaK and Its Domains

**Figure 1:**
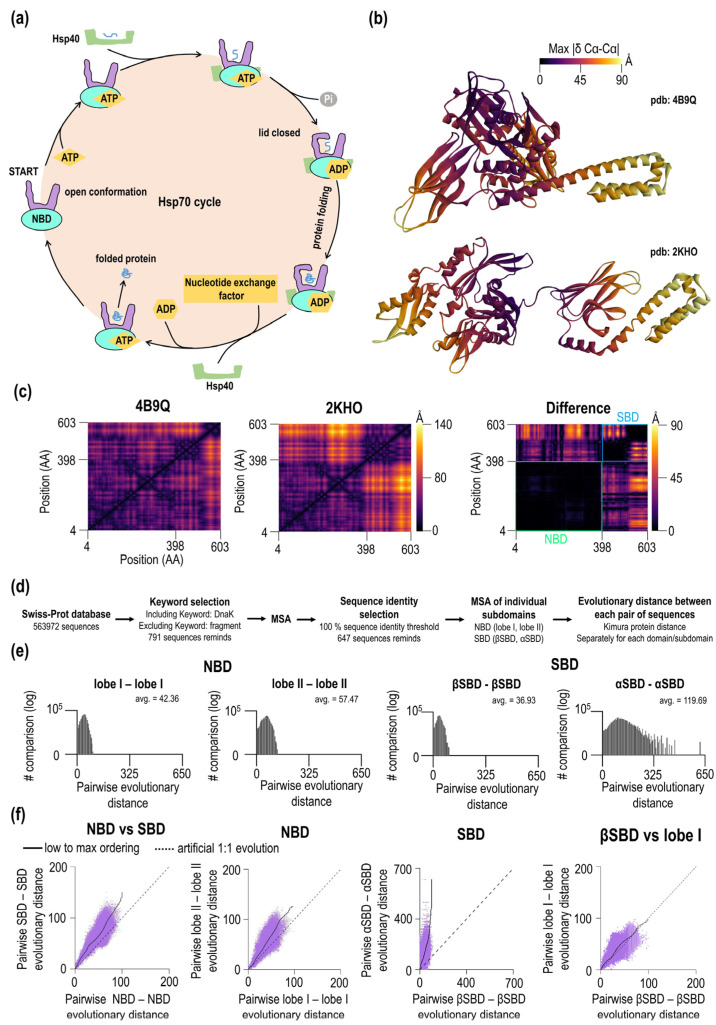
(**a**) Functional cycle of Hsp70. (**b**) Open and closed conformations of *E. coli* DnaK. The color of each residue is based on the maximum value of the difference of Cα-Cα distance (Å). (**c**) Heatmaps of Cα-Cα distances between each pair of residues in the open (avg. = 36.54 Å) and closed (avg. = 45.92 Å) conformations of *E. coli* DnaK and their differences obtained after subtraction (avg. = 14.12 Å). (**d**) Workflow of collection of sequences, selection and preparation steps for evolutionary distance analysis. (**e**) Histograms of pairwise sequence evolution distances in DnaK subdomains. (**f**) Correlation of evolutionary distances between domains/subdomains including a hypothetical equal rate of changes (dashed line).

Hsp70 proteins are two-domain proteins that undergo substantial structural changes during ATP hydrolysis (Figure 1b). The structures and phylogenetics of Hsp70 have been previously analyzed in detail. However, the potential relationship between the indels events in Hsp70 domains and phylogenesis remained unexplored and is the focus of this study.

Using three-dimensional structures of two distinct functional states, the ADP state and ATP state, we first characterized inter and intradomain contacts in Hsp70 by calculating all-against-all Cα-Cα distances (pdb: 4B9Q and pdb: 2KHO, Figure 1c—see Methods). The distance analysis found that the average distance between Cα atoms is avg. = 36.54 Å in the ATP form (SBD lid-open form), smaller than the average distance in the ADP form (SBD closed form), which is = 45.92 Å. Based on Cα-Cα distance criterion, DnaK is surprisingly more compact in the ATP form with the SBD docked and lid open conformation than the ADP form. This finding was confirmed by the analysis of other Hsp70s (Table S1). Overall, we found 15 structures, most of them in the open form and only one structure in the lid closed form (disjoined form). Consistent with our conclusion, lid open form have much smaller values of the average Cα-Cα distance than the lid closed form (2V7Y.PDB, DnaK from *Geobacillus kaustophilus*). Figure 1c shows the difference between the open and closed forms of Hsp70. The magnitude of the distance difference largely depends on the domain and the position of the given residue. There are no significant distance changes within residues in the NBD (avg. of 2.3 Å, see black areas Figure 1c in the difference heatmap). However, there are significant changes within SBD (avg. 17.04 Å) and even more dramatic changes between interdomain NBD-SBD residues (avg of. 24.75 Å). During the transition, some residues did not change their pairwise distance during open/close transition; three NBD pairs (S38—K125, G99—I338, A127—A372) and three SBD pairs (L399—P464, S493—K495, A564—L576). The largest Cα-Cα distance difference is 88.05 Å that is calculated for residue T291 (NBD) and residue T563 in SBD. In Figure 1b, we visualized the maximum value of the difference in Cα-Cα distance for a given residue for each Hsp70 conformation. When comparing subdomains, differences exist for the NBD, lobe I shows smaller average distance differences of 1.5 Å compared to lobe II with an average of 2.78 Å. Many dramatic differences are seen for SBD subdomains; for βSBD, the average difference is 1.63 Å while for the α-helical subdomain the average is 22.7 Å. Thus, lobe I and βSBD show subtle differences in their Cα-Cα distance distributions compared to the α-helical subdomain.

Having characterized differences in Hsp70 structures, we asked whether the extension of the structural changes is reflected in the phylogenesis of Hsp70. To this end, we calculated Kimura evolutionary distance from the Hsp70/DnaK protein sequences dataset, which we obtained from Swiss-Prot/UniProt (Figure 1d,e). From overall a total of 563,972 sequences, we selected 791 with the keyword DnaK in the heading and excluded sequences with a keyword “fragment” in their heading. After multiple sequence alignment (MSA) using MEGA X software [21] and MUSCLE algorithms [22] (see also “Methods”) on the set of 791 sequences, we found additional cases of identities. These sequences were removed. In the final MSA, 647 sequences remained for the analysis. The standard MSA file is available as part of Supporting Information. To focus on the folded part of Hsp70, we removed the C-terminal intrinsically disordered tail (residue 604+ *E. coli* DnaK) from the analysis and divided Hsp70 sequences into individual subdomains (lobe I, lobe II, βSBD, αSBD) according to *E. coli* DnaK numbering. Then, we performed the second MSA of subdomains and calculated their pairwise evolutionary distances (Figure 1e) The average values obtained from distribution plots for these distances are 36.93 for βSBD, 42.36 for lobe I, 57.47 for lobe II and 119.69 for αSBD. Accordingly, evolutionary distance depends on the Hsp70 subdomain with the largest pairwise distance for SBDα and smallest distance βSBD, whereas NBD subdomains have roughly similar distances. For the SBD, such significant differences in subdomain-specific values indicate large evolutionary biases within a single domain. In addition to a large distant average value, the width of the distribution of pairwise evolution distances for αSBD is quite large as well, which may be pointing to a much higher sequence adaptability to fulfill the biological function of this subdomain. 

To explain subdomain-specific distributions of evolutionary distances, two scenarios can be assumed. In the first scenario, different distributions of evolutionary distances are due to the general systematic property of functional/sequence fitness of a given subdomain. In the second scenario, observed distributions are affected by a few outliers. To test whether differences in the distributions are systematic or due to a few exceptional outliers of Hsp70, we constructed plots that correlate pairwise evolutionary distances between different DnaK subdomains. These plots show how evolutionary distances of subdomains are coupled and the continuity between the values (Figure 1f). If the amino acid substitutions within the subdomains are identical, the slope of the pairwise evolutionary distances should have a value of 1.0 (Figure 1f, thick dashed line). A correlation between the NBD and SBD (Figure 1f) shows a high degree of evolutionary coupling between domains. The SBD systematically displays a higher evolutionary distances than the NBD. Interestingly, at high evolutionary distances, the slope of the NBD-SBD correlation plot showed significant deviations from the linear dependence. For the NBD subdomains, lobe I and lobe II show a strong coupling in the evolution of these subdomains, and dependence is linear (Figure 1f, lobe I vs. lobe II). As expected, αSBD and βSBD evolutionary distances display a very high non-linear correlation between evolutionary distances. Here, the slope increases with the increasing evolutionary distance of these subdomains. A slight variation in βSBD results in a high diversity of αSBD (Figure 1f, SBDβ vs. αSBD). In the last correlation plot, Figure 1f, the subdomains with the lowest average evolutionary distance were compared, i.e., lobe I (average of 36.93) and SBDβ (average of 42.36). Here, the correlation between the evolutionary distances is strong, linear, and the slope is close to 1.0. Hence, the observed difference between NBD and SBD in the initial plot originates primarily from the variability in the evolutionary distances of lobe II and αSBD.

### 2.2. Indel Distribution from Multiple Sequence Alignment of Hsp70s

Based on the obtained multiple sequence alignment (MSA), we analyzed indel distributions in Hsp70. We also attempted to distinguish between insertion and deletion events (see also Supplementary Figure S1). These events can be distinguished based on their occurrence within the aligned sequences. The profile of insertion/deletion incidents along the alignment positions (“the master sequence”) is shown in Supplementary Figure S1. Insertions and deletions and their cumulative plot indicate that the frequency for both events is consonant, except region R75-G107 (*E. coli* sequence numbering). In this region, the number of insertion events is lower, but at position E213, the cumulative number became similar to the value for deletion events (Supplementary Figure S1). Hence, there is no preference for deletion events over the insertion events in DnaK. As mentioned before, the disordered C-terminal region was not analyzed due to gap-rich regions, making an assessment of insertion/deletion events difficult. Even though we were able to distinguish between insertion and deletion events, we grouped both events and treated them together as a sort of structural perturbation. 

**Figure 2:**
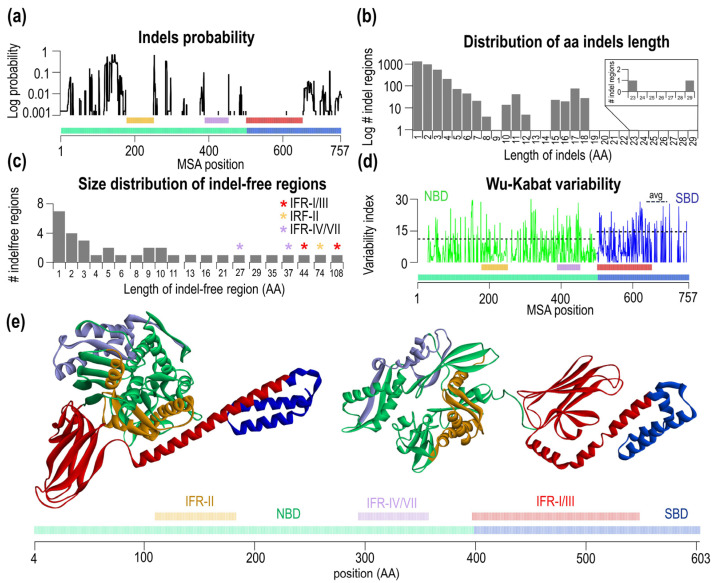
Identification and analysis of the three largest indel-free regions based on MSA of 647 sequences. (**a**) Probability of indel event for each MSA position. (**b**) Distribution of aa length of the indels in MSA (**c**) Size distribution of IFRs. The split indel-free regions are marked with asterisks. (**d**) Wu-Kabat variability of the MSA with NBD avg. variability of 11.19 and SBD variability of 14.64. The positions of MSA with a gap in the consensus sequence were not analyzed. (**e**) Open (pdb: 4B9Q), closed (pdb: 2KHO) conformations and schematic sequence of *E. coli* DnaK with the largest IFR (red) (*E. coli* numbering 397–548 aa), the second-largest (orange) (*E. coli* numbering (110–183 aa) and the third largest (purple (*E. coli* numbering 294–357 aa) indel-free regions.

In our set of sequences, there are 8886 indels in the NBD and 640 indels in the SBD, which indicates a vastly unequal distribution of indels in Hsp70 domains. When we consider the sizes of the NBD and SBD domains the 14-fold (7-fold after size-correction) difference is still remarkable. To obtain greater insights into the locations and sizes of the indels, the probability and the length distributions were calculated (Figure 2a,b). Indels are highly non-uniformly distributed along the Hsp70 sequence. In the NBD, the N-terminal lobe I region (residues ~1–180, *E. coli* sequence numbering) has many indels, which contrasts with lobe II, which shows a much lower number of indels. As expected, the SBD shows a quite low number of indels in the βSBD, and the number of indels increases significantly in the C-terminal αSBD. While the overall number of indels is high in our set, they are located non-uniformly with subdomain specificity. Figure 2b shows the distribution of the indel lengths; the number of indels initially monotonically decreases with the length until the length of eight residues. Then there are two additional clusters of lengths centered at 11 and 17 17 residues (or “aa”). The analysis can be inverted in that we analyze the sizes of regions, without indels (Figure 2c, Supplementary Figure S2). In the probability plot shown in Figure 2a, there are roughly three large regions, where indels are excluded, between res. 110–183 of *E. coli* DnaK (MSA positions 178–251) located in the NBD, 295–358 (MSA positions 389–452) in the NBD, and 397–548 (MSA positions 501–652) in the SBD. We named regions that have no indels in any Hsp70 sequence as indel-free regions, IFR, and numbered them with roman numerals according to the length in descending order (IFR-I, the largest indel-free region IFR-II the second-largest etc.). In two independent Hsp70 sequences, we found that the indel-free regions were divided by just a single amino acid insertion. In the first sequence, Hsp70 from *Desulfotomaculum reducens* contains a single T474 that splits IFR-I and IFR-III, corresponding to the position between res. 502 and 503 in *E. coli.* In the second, D328 amino acid is located in IFR-IV/VII in Hsp70 *Anaplasma marginale* (*E.coli* positions between res. 330–331). Therefore, we decided to combine both indels in the largest indel-free region of 152 aa (397–548, red bars in Figure 2, asterisks Figure 2c, Supplementary Figure S2). The overall distribution of the sizes of indel-free regions is shown in Figure 2c and Supplementary Figure S2. With the increasing length of indel-free regions, the frequency of their occurrence gradually decreases until the length of four residues. IFR displays various sizes, mostly clustered around certain values (see values on the *x*-axis, Supplementary Figure S2. 

Next, we asked whether the IFRs have different variabilities or, in other words, whether there is a relationship between variability and the indel-free character of the given region. To this end, we analyzed Wu-Kabat variability, shown in Figure 2d NBD green, SBD blue. There are differences in average Wu-Kabat variability between NBD (value of 11.19 ± 8.11) and SBD (14.64 ± 8.39). IFR-II has a variability of 7.95, IFR-IV/VII of 13.88 and IFR-I/III variability of 12.01. Thus, we concluded that IFRs do not constrain their amino acid variability. These IFR regions have conserved the length, and have not conserved their amino acid composition. By highlighting three large IFRs in the structure of ATP and ADP form of DnaK (Figure 2e), the two largest IFRs shown in red/orange are the part of the NBD/SBD interface in the ATP state, however they are far away from each other in the ADP conformation. 

### 2.3. Phylogenesis of Hsp70 in Firmicutes and Proteobacteria

**Figure 3:**
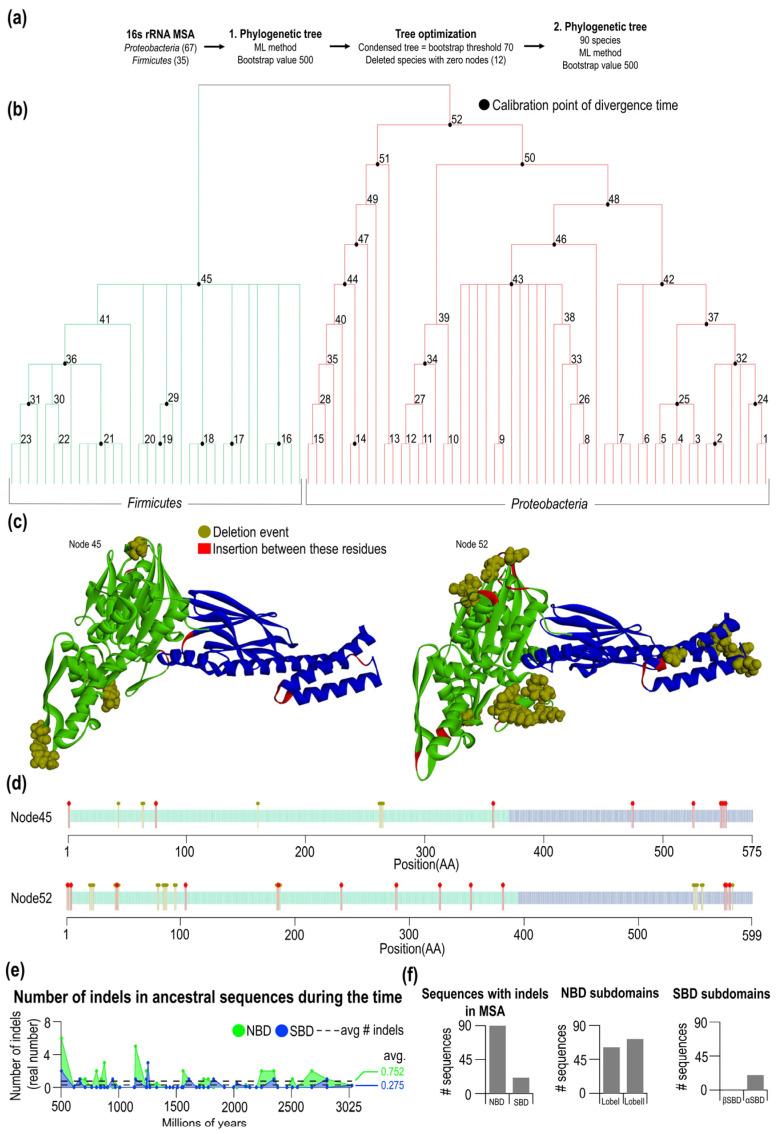
Condensed 16S rRNA phylogenetic tree of *Proteobacteria* and *Firmicutes* and indels evolution in ancestral sequences. (**a**) Workflow of the 16S rRNA phylogenetic tree construction steps. (**b**) The condensed form of the 16S rRNA phylogenetic tree of *Proteobacteria* and *Firmicutes* was constructed by the maximum likelihood method with 52 nodes. Black circles represent calibration points, from which the divergence time was calculated. (**c**) DnaK predicted structures of the oldest ancestral sequences for both *Proteobacteria* (node 52) and *Firmicutes* (node 45). Positions where the indels occur during analyzed evolution are colored red (insertions) and brown (deletions). (**d**) Schematic sequences of the oldest ancestral sequences of *Proteobacteria* and *Firmicutes* with colored indels positions. (**e**) Number of indels in *Proteobacteria* and *Firmicutes* ancestral DnaK domains (NBD avg. = 0.752, SBD avg. = 0.275) during the evolution. The indels ratio for every node is divided by the number of sequences in node-1. (**f**) The number of identified indels in the present domains and subdomains of *Proteobacteria* and *Firmicutes* DnaK sequences.

To analyze how indels are formed in evolution, we conducted ancestral sequence reconstructions. Notably, the other selection is needed to reconstruct the phylogenetic tree reliably. Hence, we conducted a deeper manual selection of sequences (see Supplementary Materials, Supplementary Figure S3 and Table S2). We selected bacterial sequences belonging to members of the two most frequent bacterial phyla—*Proteobacteria* (67 species) and *Firmicutes* (35 species), which resulted in a new set of 102 cured sequences. To construct a maximum-likelihood phylogenetic tree, 16S rRNA were collected, aligned and used as input for the calculation (Figure 3a). From this step, the first phylogenetic tree (Supplementary Figure S4) was calculated with 500 bootstrap replicates. Subsequently, a condensed tree was calculated with a bootstrap threshold of 70%. After this step, 12 sequences did not have any node and were therefore deleted (Supplementary Figure S5). A new phylogenetic tree was calculated from 90 sequences (Supplementary Table S3), and its condensed form is shown in Figure 3b. In this condensed tree, there are 52 nodes at different tree depths. For each node, we calculated an ancestral sequence (see Methods). For the two oldest sequences at nodes 45 and 52, we predicted the three-dimensional structure of these ancestral proteins using Robetta from David Bakers lab [23]. We calculated the average error of the predicted 3D structures from errors at each position. The calculation of error per residue is based on coordination distances (in Å) of residues Cα of predicted and native structures. The overall error of the model is 0.69 Å and 0.75 Å for nodes 45 and 52, respectively. The reliability of the structural determination at arbitrary residue, the reader is referred to Supplementary Figure S6. For each ancestral sequence, we tracked insertion and deletion events in *Firmicutes* (node 45) and *Proteobacteria* (node 52). This tracking made it possible to analyze indel events in Gram-negative and Gram-positive phyla. In Figure 3c,d, indel events are mapped into 3D and primary sequence of node 45 and node 52 ancestral proteins. For node 45, there are 15 NBD and 6 SBD indel events. In the NBD of node 45, we found three regions where insertion occurred and eight positions where deletion events occurred. Only insertions were observed for the C-terminal region of NBD (res. greater than 357–358), while indel-free region IFR I/III is preserved. As seen in the 3D model of node45, deletion events occurred at the outer surface parts of the protein, as observed in general. Insertions are observed in the loops connecting secondary structure elements. For node 52, there are 38 NBD and 12 SBD indel regions. In the NBD of node 52, we found nine regions where the insertion occurred and 24 positions where deletion events occurred. In the SBD of node 52, again, the indel-free region IFR I/III is preserved, and at the C-terminal end of the SBD, we found both insertions and deletions (Figure 3c,d, node 52). 3D structural mapping of indel regions revealed that insertion and deletion occurred within structural elements. 

Using ancestral reconstruction and calibrated divergence times (Supplementary Tables S4 and S5), we tracked the number of indel events over time for NBD and SBD (Figure 3e). Most of the time, indel events predominate in the NBD over time with an average of 0.75, and for the SBD, we found 0.28 on average. The number of events slightly decreases toward the past due to the number of available sequences decreasing. The length of ancestral domains remained relatively constant after *Firmicutes* and *Proteobacteria* separated, and no sign of the length convergence is seen or any systematic reduction in the domain lengths. Notably, the largest indel event occurred before the division between Firmicutes and *Proteobacteria*. In node 45, a part of the sequence is missing in the NBD, present in *Proteobacteria*—node 52.

From the MSA set of 90 selected sequences from *Firmicutes* and *Proteobacteria*, we found in all NBD sequences indels while for the SBD, only 21 indels were identified (Figure 3f). 

A roughly equal number of the indels in the NBD are distributed between lobe I (found in 61 out of 90 sequences) and lobe II (found in 72 out of 90). In the SBD, as expected from the previous analysis, indels are in αSBD (20 out of 90 sequences) and one sequence in the βSBD—Hsp70 from *Desulfotomaculum reducens*. 

### 2.4. Amino Acid Residues Participating in Interdomain Contacts Are Frequently Located in Indel-Free Regions

Based on the phylogenetic analysis, we found that indel events are similarly localized for *Firmicutes* and *Proteobacteria*. At the same time, the NBD and SBD domain show relative constant domain sizes, which supports our previous speculation on the importance of the physical orientation of domains in the ATP form. The orientation of the domains is stabilized by ATP-specific interdomain contacts, which we define as the distance between Cα-Cα less than 7 Å in the open form. Based on this definition, we found 33 contacts and contacts with less than 2 Å difference between open and closed forms were removed. 

**Figure 4:**
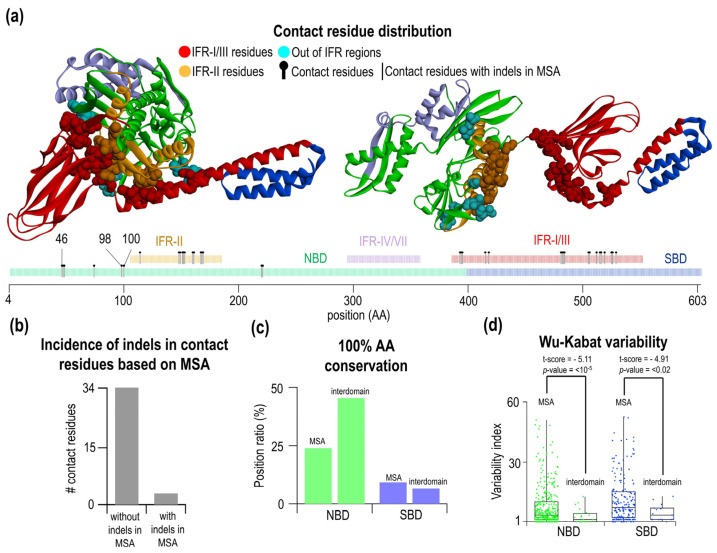
Characterization and analysis of contact residues. (**a**) Visualization of contact residues in the open (pdb: 4B9Q), closed (pdb: 2KHO) conformations, and schematic *E. coli* DnaK sequence. The contact is defined as a Cα-Cα distance < 7 Å of contact residues and with their conformation differences more than 2 Å. Totally, they are 37 residues in contacts. In three specific residues, a gap was found at the corresponding MSA positions. (**b**) Number of interdomain residues in the regions without or with indels. (**c**) Comparing of aa conservation of contacting interdomain residues with the whole MSA. The overall conservation of the NBD is 45.46% in interdomain contact residues compared to 24.01% in the whole MSA. (**d**) Comparing of Wu-Kabat variability of the interdomain residues in the NBD/SBD with the whole MSA. The average Wu-Kabat variability of MSA is 8.31% for NBD and 11.73% for the SBD. Interdomain contact residues had variability of 3.62% in the NBD and 5.33% in the SBD.

Finally, we arrived at 31 contacts. These contact pairs are formed by two amino acids of overall 22 NBD residues and 15 SBD residues (Figure 4a, see Table 1). One residue can have multiple contacts to other residues. These contacts are formed by one interacting partner from the NBD and the other from the SBD. Interacting residues are localized along the Hsp70 sequence (Figure 4a).

Interestingly, interdomain contact pairs are found at high frequency in large indel-free regions. As mentioned before, three IFR can be identified in the Hsp70 (red IFR-I/III, orange IFR-II, and light purple IFR-IV/VII). Interdomain residues are located only in the two largest IFRs, IFR-I/III in the SBD and IFR-II in the NBD. The region IFR-IV/VII does not contain any interdomain residue and is not involved in the allosteric change from open to a close form of Hsp70. Aside from large IFR, a minority of interdomain contacting residue is located in the smaller indel-free regions. There are eight residues outside of large IFRs, and three out of these are located within a position in the MSA where indels happened. Figure 4b summarizes this result—34 residues are in IFR, and three are in the indel positions. Next, we analyzed the level of conservation of the residues forming interdomain contacts from the NBD Figure 4c. We found that these contacts contain more conserved residues, 45%, than 24% conserved residues on an average position in the MSA. This is not observed for the SBD residues involved in the interdomain contacts; a roughly similar (6.67%) number of conserved residues are present as on MSA average (9.31%). This finding is surprising and may indicate that SBD residues must not be conserved to pair with the NBD residues. We wondered whether the variability of these residues is affected and hence some preference for the conserved NBD residues at all. Using our new dataset of 90 Hsp70 sequences, we calculated Wu-Kabat variability (Figure 4d). As expected, the NBD Wu-Kabat variability for ATP-specific interdomain residues was lower than on average (MSA—8.31; interdomain—3.62). In the SBD, we found that the variability of the residues is much lower than on average (MSA—11.73; interdomain—5.33). This indicates that to contact conserved NBD interdomain residues, the SBD residues do not need to be conserved; however, their variability decreased, indicating rather partial specificity of the interdomain SBD residues. We validated the significance of the observed differences between variability interdomain contacts vs. intradomain residues by the Welch’s *t*-test, which takes into account different sample values and different class variance (Figure 4d, for the NBD *p* < 10^−5^ and for the SBD *p* < 2 × 10^−2^). 

### 2.5. Clustering of Interdomain Residues Using Principal Component Analysis and Kernel Density Estimation

**Figure 5:**
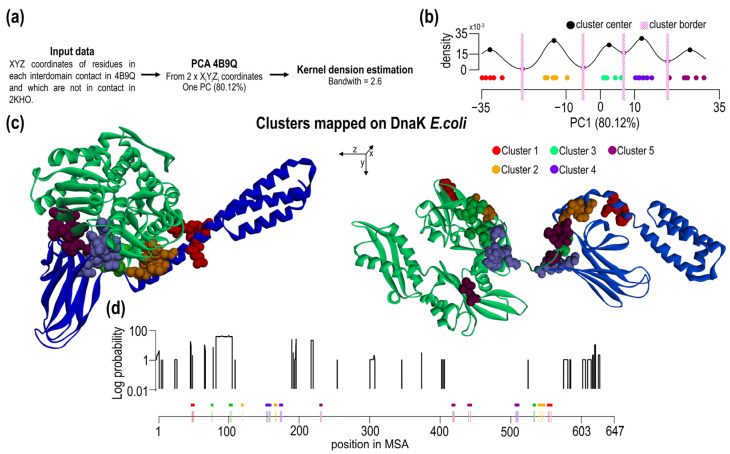
Clustering of contact residues. (**a**) Workflow of cluster analysis. (**b**) Kernel density estimation of PC1 data (see also Supplementary Figure S7). Based on density, we found five clusters of contact residues. (**c**) 3D structures of open and close conformation of *E. coli* DnaK with visualized clusters. Structures are aligned across the *z*-axis. In this axis are residues distributed. (**d**) Probability of indels in positions of *Proteobacteria* and *Firmicutes* MSA. Schematic MSA positions show the distribution of contact residues in MSA with corresponding clusters.

Based on previous findings, one may conclude that interdomain contacts may or may not contain conserved residues and hence the contacts can have different contribution to the interface. We attempted to sub-divide contacts based on their spatial/geometrical properties and correlate with the presence of conserved residues. To this end, we analyzed pairs of residues forming overall 31 contacts using principal component analysis followed by the analysis of the existence of “pair clusters” by kernel density estimation (Figure 5a). Based on the PC1 values, there is a clear tendency of some residues to be grouped around specific PC1 values, i.e., there is increased local density of values. Such agglomerates of 1D points we termed as clusters and analyzed accordingly. Using kernel density estimation, five clusters have been identified, and they are well separated from other density points (Figure 5b). Residues belonging to different clusters and conserved residues are summarized in Table 1. Contacts and residues belonging to different 1D clusters are shown in Figure 5c in the open and closed form of Hsp70. 

The type of clusters shows spatial correlation and their different sensitivity toward evolutionary changes. A set of the interdomain contacts and the clustered residues were correlated with the probability of indel events (Figure 5d). Obviously, all SBD residues forming interdomain contacts are located in IFR-I/III of all clusters. In the NBD IFR-II, on the other hand, only clusters 2 + 4 are found. The other contacting residue in the NBD 2 residues (98, 100 *E. coli* numbering) of cluster 3 and 1 residue of cluster 1 (pos46G) are in the position where indels are found in Hsp70s.

## 3. Discussion

### 3.1. The Evolutionary Rate of Structurally Distinct Subdomains of Hsp70 Are Considerably Different

Hsp70 is a molecular ATP-dependent chaperone consisting of two distinct folded domains—the nucleotide-binding (NBD) and substrate binding domains (SBD). Each domain can be further divided: the NBD consists of lobe I and lobe II whereas the SBD consists of β-SBD core and α-helical subdomain. Each subdomain plays a different role in molecular action during the Hsp70 allostery. The individual roles of the subdomains for allosteric coupling are highlighted in the 3D structures of different Hsp70s in open and closed forms [24,25,26]. The crystal structure of an intact ATP-bound Hsp70 from *E. coli* shows that ATP-bound NBD adopts a unique conformation, forming extensive interfaces with an SBD, which has αSBD opened and restructured βSBD. The existence of extensive domain-domain interfaces indicates that indels can be highly disruptive predominantly due to a collective shift between interacting pairs of residues. The importance of a fine local mechanical balance between Hsp70 substructures can be seen in 3D structures of an intact human BiP (Hsp70 in ER) in an ATP-bound state [24]. In this structure, the authors found that peptide binding loops in the SBD moves dramatically and their re-arrangements result in a full closure of the grasping loops forming the peptide binding pocket. Hence, in this ATP-bound form, the localized structural changes in the SBD exclude any substrate binding and point out a dynamic nature of the polypeptide-binding pocket in the Hsp70 chaperone cycle. Such dynamics of loops may provide additional clues why indels are avoided for the correct functioning. Additionally, post-translational modifications (PTMs) can impact the Hsp70 chaperone cycle [27]; here, indels may change the PTM profile and, hence, modify the function. 

In this study, we find highly different evolutionary distances between domains and subdomains of Hsp70, which indicate they explore structure-specific evolutionary fitness landscape sizes. At the level of structural domains, NBDs showed smaller evolutionary distances compared to the SBD, which might be due to functional conservation of the ATP-binding site. We speculated that higher evolutionary distances for SBD are due to functional reasons as well because of the broad substrate specificity of the SBD; it binds several hundred protein clients [28]. As bacterial protein clients undergo evolutionary changes as well, SBD may be forced to adapt its structure by exploring sequence space more extensively compared to functionally more conserved NBD, which, in turn, would explain larger SBD evolutionary distances. However, this speculation was not supported by our more detailed analysis of the Hsp70 substructures. Namely, the substrate binding pocket of the SBD is highly conserved as opposed to the α-helical part, and hence, a high variability of the SBD is due to subdomain that is not directly involved in the peptide binding. 

NBD lobe I and lobe II show significant differences in their pairwise evolutionary distances; lobe II-lobe II evolutionary distances are greater than for lobe I-lobe I. In previous publications we showed that lobe II and lobe I have vastly different mechanical stabilities, and lobe II acts as a folding nucleus that has a native fold and the ability to bind nucleotides [29]. It was estimated that the total number of stable proteins is very small and it anti-correlates with the protein length [30]. Therefore, a stable mini-domain (lobe II NBD) may be more conserved than unstructured subdomain (lobe I NBD) because a possible sequence diversity is much larger for the latter. However, no correlation was found between mechanical stability of subdomains and evolutionary distances. The reason is that the formation of the subdomain interface between lobe I and lobe II, for example, limits the sequence diversification. At the same time, the binding specificity for the ATP versus ADP nucleotide originates in lobe I that coordinates the positioning of the magnesium cation and the terminal γ-phosphate group. As a result, lobe I is also involved in the transmission of allosteric changes due to conformational changes in the subdomain Ia-Ib orientation of lobe I. In the NBD, lower evolutionary distances for lobe I indicate the presence of additional functional constraints that preserve the conformational changes during the phylogenesis—intrinsic stability is not crucial unless lobe II take over the role of stabilizing entity. 

In the SBD subdomains, we observed even more dramatic differences between pairwise distances of the subdomains. SBD-β has much smaller pairwise distances compared to α-helical subdomain. This difference is so remarkable that it can be described as a sizeable evolutionary asynchrony between the tightly packed subdomains in a single folded domain. For the SBD, we also observe no obvious correlation between substructures of different mechanical stability that were previously investigated [31]. Similar to the NBD, we can exclude that different mechanical stabilities of subdomains are the cause of the variability in the evolutionary distances. At the same time, βSBD is highly conserved. SBD evolutionary distances are highly correlated to the lobe I evolutionary distances. The slope of this correlation is close to 1.0, indicating a similar evolutionary diversity rate. In contrast, the α-subdomain of the SBD has the highest variability among Hsp70 subdomains. Such highly variable substructures potentially play only a role in the structural integrity and are not involved in the conserved allosteric interdomain contacts. Hence, the structural role of α-helical domain can be fulfilled by a large number of sequences that can fold into stable α-helical bundles. 

### 3.2. Non-Random Distributions of Indel-Free Regions in Bacterial Hsp70s

During the analysis of multiple sequence alignment (MSA) of Hsp70, we found that the probability of an insertion/deletion event is not uniform along the polypeptide sequence. We also found some very long regions with no indels and called these uninterrupted regions indel-free regions (IFRs). From the absence of the indels, we infer that indels in these regions are highly disruptive, possibly due to functional or stability reasons. The spectrum and distribution of indels might be viewed as traits specific to the evolution of Hsp70. While amino acid substitutions and correlations between distal sites have been analyzed in detail and are accepted due to species’ evolution and variability, indels were not previously analyzed at the same depth. One reason might be that indels are less frequent, and identification of small indels might be complicated and somewhat arbitrarily dependent on algorithms and the parameters used for the MSA. In particular, small indels might be challenging to localize accurately due to natural sequence variability. In our MSA, we focused on long IFRs that can be identified reliably due to their localization within conserved regions of lobe II and β-SBD. Hence, the IFRs identified in our study are well-defined due to the presence and positioning of several conserved residues. In some cases, large IFRs were split by a single amino acid in a single bacterial species. We decided to consider such events as very rare, and therefore in such cases, we fused those IFRs. 

In the evolution of proteins, the presence of conserved residues is often viewed as an indication of their functional importance. Here we extend this by pointing out the same for the presence of large indel-free regions. The presence of highly conserved residues can be intuitively associated with some crucial contributions to biological functions, e.g., nucleotide binding, catalytic action, allosteric signaling and others. However, the existence of large indel-free regions in proteins may not be so intuitive. Conserved IFRs generate a constant physical distance between distal sites. Assuming large conformational changes in Hsp70 and changes in the interacting interfaces, one can associate the constancy of the physical length to the pairing of a particular part of the subdomain to two different parts of the Hsp70 structure. In the closed ADP form, the α-subdomain interacts exclusively with β-SBD. In the open ATP-form, however, the α-subdomain interacts with lobe I. An insertion or deletion event in these substructures inevitably lead to a global shift between paired residues and, hence, a large mismatch between subdomain contacts. For a single interface, amino acid substitutions may compensate for such a shift. In contrast, the presence of two different subdomain interfaces (one with SBD and one with lobe I) would require several correlated mutations to compensate for such shift, which is less likely. To determine whether IFRs reflect a truly independent evolutionary bias, we analyzed the IFR’s amino acid conservation variability and found no difference between IFR and regions with indels (see Figure 2d). 

In total, three large IFRs were detected; two of them, IFR-II and IFR-I/III, are located close to each other in the ATP-form of Hsp70, but in the ADP-form. Clearly, these two IFRs are part of the interdomain interface specific to the ATP-form. In addition, IFR-I/III contains elements of the structure in the SBD that create intradomain interactions and hence are part of the α/β interface in the closed ADP form of Hsp70. The third, IFR-IV/VII (purple Figure 2e), is located on the other side as a part of lobe II and is not in contact with any IFRs in any form of Hsp70. The IFR-IV/VII connects two sub-structures of lobe II, which includes the nucleotide-binding cavity. Here, the physical distance between lobes IIa and IIb may help to define the correct positioning of the residues that coordinate and bind nucleotides. 

To gain more insight into the fate of indels during Hsp70 evolution, we reconstructed the phylogenetic tree (Figure 3b). An accurate tree can be reconstructed when enough members of the phyla are collected. Here, we reconstructed and compared *Firmicutes*—Gram-positive bacteria phyla and *Proteobacteria*—Gram-negative phyla. Using these two phyla, the ancestral sequences can be obtained and analyzed for the presence of indels over time. We found that during the evolution of these two phyla, the most significant indel events occurred very early in history, i.e., before they diverged. Gram-positive/negative differences in Hsp70 insertion has previously been observed [15,16]. During the evolution of *Firmicutes* and *Proteobacteria*, only minor changes and adjustments were observed compared to before they diverged. For both phyla, we found a distinct spectrum of indels; indels in lobes I and II are roughly similar yet not identical. Again, the domain-specific frequency of indel events was observed in ancestral sequences. On average, three times more indels were detected in the NBD than in the SBD. Over time, small indel events were frequently identified in evolutionarily younger species, and no indication of the saturation was identified. 

### 3.3. Interdomain Contacts Tend to Be Located within Indel-Free Regions

Combining MSA and structural analysis, we find that most interdomain contacts are located in IFR-I/III and IFR-II. A very few interdomain contacts are outside of these indel-free regions. Here, we concluded that overall sequence variability in IFRs is similar to that in other regions. When looking specifically at interdomain contact residues within IFRs, we found that NBD contacts contains more conserved residues, whereas SBD residues show the same amount of conserved residues. This finding is consistent with the NBD being more conserved and having lower variability compared to the SBD. It indicates that some contacts from the NBD are formed by conserved residues that are paired with non-conserved residues from the SBD. Hence, interdomain residues may be non-equivalent and contribute differentially to subdomain interfaces. To carry out a deeper analysis and distinguish between various contacts, we developed and applied a more general workflow that combines a principle component analysis of structural coordinates of unique interdomain pairs (that exist only in the open form) and then analyzed clusters in the PCA plots. This analysis provides information on the nature of structural changes, the most variable axes and the overall magnitude and directionality of the structural changes. All SBD contact residues and the majority of the NBD contact residues are located in indel-free regions. Only a few NBD residues are located outside of the IFR and may not constitute a conserved core subdomain interfaces. At the same time, a number of conserved residue contacts are distributed heterogeneously over space. Categorizing contacting residues into clusters may help to differentiate between contributing clusters to stabilize the ATP-form of Hsp70. Using PC1, we found five clusters, which contain different numbers of conserved residues and may indicate their unequal importance and contribution to the subdomain interface. Cluster 1, for example, does not contain any conserved residue, while clusters 4 and 5 contain four conserved residues, which may imply greater importance in preserving ATP-specific subdomain interfaces. Moreover, cluster 4 also includes a single conserved contacting residue from the SBD. Hence, our approach enables researchers to identify clusters that contain different evolutionary-conserved residue contacts. 

### 3.4. Hsp70 Subfamily Member HscC Contains Indels in IFR-I/III

In Gram-negative *Proteobacterium E. coli*, aside from heat-shock regulated Hsp70 DnaK, two additional Hsp70 homologs exist: HscA (38.4% of sequence is identical to DnaK) and HscC (26.7% of sequence is identical to DnaK) [12]. HscA is involved in assembling iron sulfur clusters [32]. HscC participates in resisting Cd2+ ions and UV-light [33]. Interestingly, both HscA and HsC contain several insertions and deletions in their NBD and SBD [34]. Hence, indel events do not necessarily functionally compromise the structure of the domains. In HscA, particular sites of insertions and deletions do not contain residues that form inter-domain contacts during ATP-induced allosteric transitions. In HscC, closer inspection reveals few insertions and several medium-sized deletions in IFR-I/III. Kluck et al. [33] studied biochemical properties of HscC in vitro and in vivo and found that HscC does not show ATP-dependent binding/refolding activity on client protein substrates. At the same time, the temperature sensitive phenotype of *ΔdnaK52* strain (BB1553) cannot be supplemented by even overexpressed HscC. We speculate that allosteric regulation as seen in DnaK is highly perturbed in HscC, which may be due to deletions in the SBD‘s IFR region.

## 4. Materials and Methods

For the analysis, we used DnaK protein sequences from the UniProt (https://www.uniprot.org, accessed on 20 January 2022) database, UniProtKB/Swiss-Prot release 2020_06. The database contained 563,972 sequences. With keyword: DnaK in sequence head and after exclusion of fragments of Hs70 sequences there were 791 sequences. After exclusion of identical sequences, the set contained 647 Hsp70 protein sequences (alignment can be obtained on request or on publicly available repository). The 16S rRNA sequences of the bacteria, from which the DnaK sequences were analyzed were obtained from the SILVA SSU r138.1 database (https://www.arb-silva.de, accessed on 20 January 2022). As a model 3D structures in this article we used the *E.coli* DnaK open (pdb: 4B9Q, [34]) and close (pdb: 2KHO, [5]) conformations, which were visualized in Discovery studio 2019 (BIOVIA, Dassault Systèmes, Discovery Studio, Dassault Systèmes: San Diego, CA, USA, 2019).

DnaK protein and 16S rRNA alignments were calculated in MEGA X software [21] using MUSCLE algorithms [22]. For RNA alignments following parameters were used: gap open: −400, gap extend: 0 and the UPGMA clustering method, and for protein MSA, following default parameters were used: gap open: −2.90, gap extend: 0.00, hydrophobicity multiplier: 1.20, and UPGMA clustering method. According to *E. coli,* we divided the DnaK MSA into the domains NBD (1–398), SBD (399–603) and C-terminus (604+). Then we did MSA separately for each domain and manually reviewed aligned positions.

The sequence identity matrix [35] and Wu-Kabat variability (Equation (1)) [36] were calculated as described previously [37]. Wu-Kabat variability index describes the amino acid variability in each position of the alignment to the alignment consensus sequence. *Variability index* was calculated by following equation:

(1) $$variability\;index=\frac{N\times k}{n}$$

where *N* represent the total number of sequences in the alignment, *k* is the number of different amino acids at a given position, and *n* is the frequency of the most occurring amino acid in that exact position. To calculate MSA indels probability distribution, we started with the determination of indel probability at each alignment position. This probability was determined as the indels ratio compared to the number of “sites” (sequences) at the analyzed MSA position. Each “site” in MSA positions that were identified as gaps in the consensus sequence was not included in calculation of Wu-Kabat variability (i.e., “sites” where the gap represented only the insertion space in another sequence). The decision of position identification as insertion or deletion was made by gaps occurring in every specific position. Positions with more than 50% of gaps were defined as insertions.

The Cα-Cα distances were calculated from model structures in the Chimera 1.15 software [38]. In our case, the contact was defined as a Cα-Cα distance less than 7Å in open (PDB: 4B9Q) *E. coli* DnaK conformation. The second condition was a distance difference of the same Cα-Cα between both conformations must be more than 2Å.

For calculating pairwise evolution distance matrix, we used Kimura protein distance [39] as a part of EMBOSS tool Distmat (Equations (2)–(4)) [40]. In this method, gaps are ignored, and the formula is:

(2) $$S=\frac{m}{npos}$$

(3) D=1−S

(4) $$distance=-\ln\left(1-D-0.2\times D^{2}\right)$$

where *m*—exact match, *npos*—number of positions.

To determine the best-fit substitution model [39], we compared 56 models of amino acid substitutions for each DnaK domain separately. The algorithm used the maximum likelihood statistical method and 16S rRNA phylogenetic tree as a template. We considered the model with the lowest BIC score (Bayesian Information Criterion) to be the best, and all models were calculated using MEGA X software.

A phylogenetic tree was calculated using 16S rRNA sequences by the maximum likelihood method [41]. We used Kimura 2 parameter substitution model G + I with bootstrap replication set to 500. The condensed phylogenetic tree was calculated based on a bootstrap value threshold higher than 70%. The tree was calculated using MEGA X software. For the bacteria classification, we used the NCBI taxonomy database. (https://www.ncbi.nlm.nih.gov/taxonomy, accessed on 20 January 2022). 

For estimation of species divergence time, we used the RelTime-ML method [42,43], Kimura 2 parameter model and the 16S rRNA phylogenetic tree. To set the borders for minimum and maximum species divergence time we took, the estimations from timetree.org knowledge-base [44]. Timetree collecting data about species divergence were from publications. For better relevance, we took only those estimates from at least 3 publications and analyzed only nodes of the condensed 16S rRNA phylogenetic tree (where the bootstrap value was greater than 70%). This calculation was performed twice, separately for *Proteobacteria* (16 calibration nodes) and separately for *Firmicutes* (9 calibration nodes). For each calculation, the second strain was set as an outgroup.

As a template for estimating an ancestral sequence, we used DnaK protein sequences and a 16S rRNA phylogenetic tree. A substitution model with the lowest BIC score was included in the calculation, and the sequences themselves were calculated by the maximum likelihood statistical method and the Le Gascuel model [45]. We estimated the ancestral sequences only for the nodes of the condensed tree. The most probable ancestral amino acid at each position is given the highest probability among all possible amino acids. Ancestral sequences were calculated using MEGA X software.

Ancestral protein structures were calculated using the RoseTTA fold method [46] in the Robetta protein structure prediction server (https://robetta.bakerlab.org, accessed on 20 January 2022). PCA analysis was provided by the KNIME analytics platform (https://www.knime.com, accessed on 20 January 2022). Kernel density estimation with bandwidth optimization was provided by Web Application for Kernel Bandwidth Optimization (Ver. 0.4) [47]. Statistical significance of Wu-Kabat variability (Figure 4d) was calculated using Welch’s *t*-test [48]. For other data analysis we used the following Python libraries: Biopython (https://github.com/biopython/biopython, accessed on 20 January 2022), Pandas (https://github.com/pandas-dev/pandas, accessed on 20 January 2022), Numpy (https://github.com/numpy/numpy, accessed on 20 January 2022). Heatmaps were visualized by the seaborn library (https://github.com/mwaskom/seaborn, accessed on 20 January 2022). 

## 5. Conclusions

In the presence of ATP, Hsp70 undergoes large conformational changes in the SBD domain and α/β-subdomains are separated from each other. This large rearrangement of the Hsp70 structure results in new domain-domain interactions. Here, we provide compelling evidence that residues involved in inter-domain contacts and thus creating an allosteric interface are preferably located in indel-free regions. The plausible explanation is that productive interactions between allosteric interfaces provide tight spatial restrictions. The length change due to indel events disrupts the pairing of inter-domain interactions for which mutations cannot easily compensate. We conclude that designing allosteric machines requires a fine mechanical balance between protein-protein interfaces.

## Figures and Tables

**Table 1:** *E. coli* Hsp70 contacts within each of identified clusters. * conserved residue NBD, ** conserved residue SBD.

Cluster ID	The Number of Conserved Residues NBD + SBD	All in IFR	Contact Residues (*E. coli* Numbering)
1	0 + 0	No	G46-A525, E47-A525, E47-D526, E47-F529, T48-D526
2	1 + 0	Yes	Q114-M515, * I160-I512, * I160-M515, * I160-V516, * I160-A519, A161-V516, A161-A519
3	2 + 0	No	* G74-G506, N98-S505, D100-S505, 149-S505, A149-G506, * A153-G506
4	4 + 0	Yes	* D148-I483, * D148-L484, * R151-I483, Q152-I483, * R167-D481, * I168-D481, I169-D481
5	3 + 1	No	A220-N415, * T221-N415, * D393-I418, * V394-I418, T395-I418, T395-** G482

## Supplementary Materials

The following supporting information can be downloaded at: https://www.mdpi.com/article/10.3390/ijms23052788/s1.
