# Supplementary material for: Allosteric Inter-Domain Contacts in Bacterial Hsp70 Are Located in Regions That Avoid Insertion and Deletion Events
Source: Int J Mol Sci. 2022 Mar 3;23(5):2788. doi: 10.3390/ijms23052788 (PMC8910936; doi:10.3390/ijms23052788)
Supplement: Supplementary file 1 [file ijms-23-02788-s001.zip › ijms-1594067-supplementary.pdf]

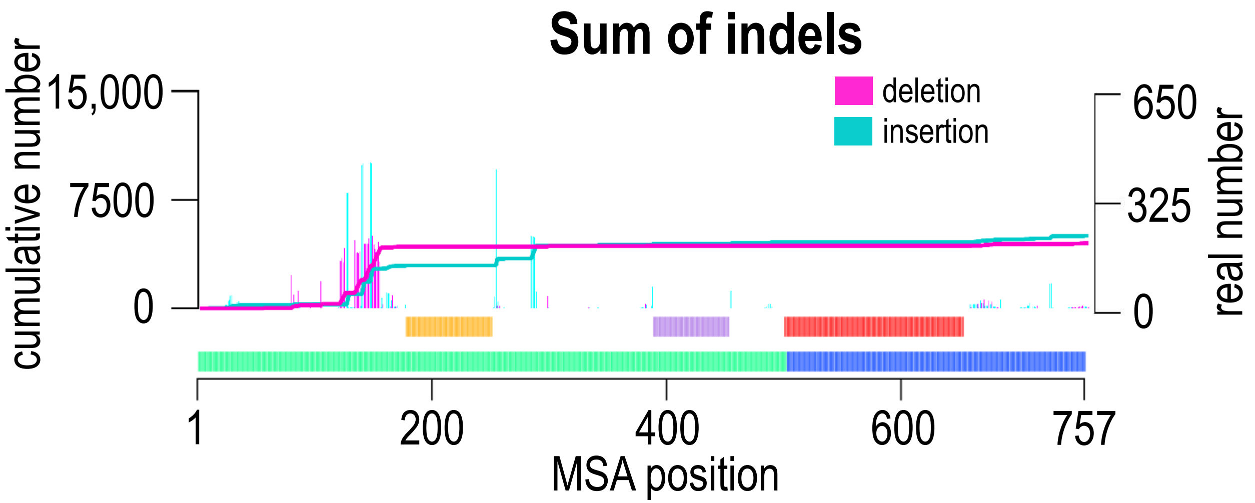

Figure S1. Cumulative sum of indels with real number of indels.

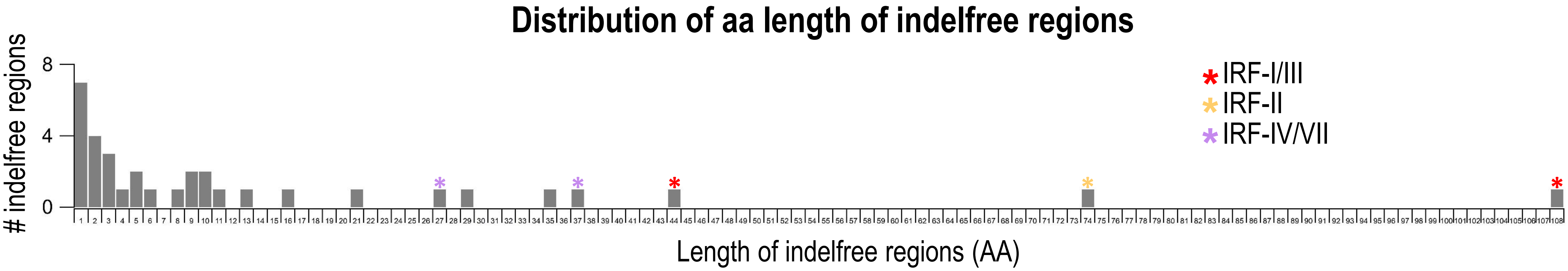

Figure S2. Various size distribution of indel-free regions.

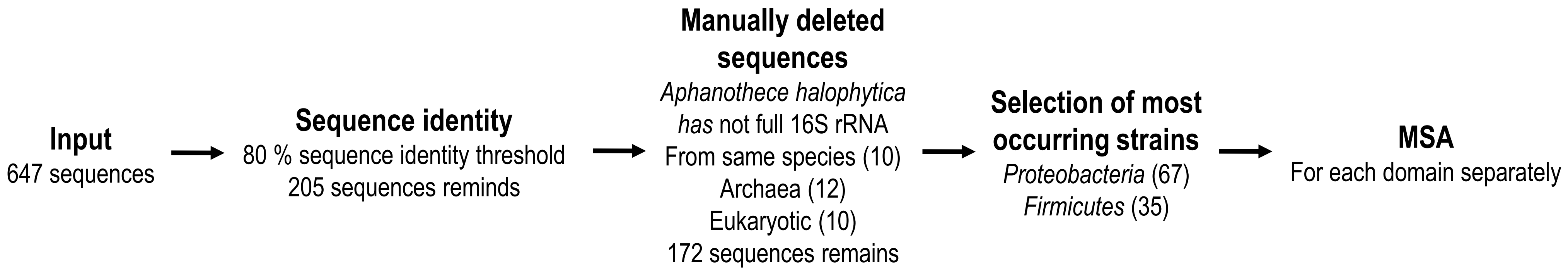

Figure S3. Workflow of sequence selection steps and choosing final taxons.

Table S1. Average Cα-Cα residue distance in selected Hsp70 proteins.

Strain	Pdb	Form	Method	Average Cα-Cα distance (Å)	References (doi)
Escherichia coli	7KO2	Restraining state	X-RAY DIFFRACTION	36.49	10.1016/j.molcel.2021.07.039
Escherichia coli	7KRT	Restraining state of a truncated DnaK	X-RAY DIFFRACTION	36.65	10.1016/j.molcel.2021.07.039
Escherichia coli	5NRO	J-domain bound	X-RAY DIFFRACTION	36.78	10.1016/j.molcel.2017.12.003
Escherichia coli	4JNE	Allosteric opening	X-RAY DIFFRACTION	36.97	10.1038/nsmb.2583
Escherichia coli	7KRW	Stimulating state fused with a substrate	X-RAY DIFFRACTION	39.71	10.1016/j.molcel.2021.07.039
Escherichia coli	7KRV	Stimulating state of disulfide-bridged	X-RAY DIFFRACTION	34.31	10.1016/j.molcel.2021.07.039
Escherichia coli	7KZI	Intermediate state (QQQ)	X-RAY DIFFRACTION	38.46	10.1107/S2059798321002436
Escherichia coli	7KZU	Quasi-intermediate state (Q) of a truncated Hsp70 DnaK	X-RAY DIFFRACTION	31.99	10.1107/S2059798321002436
Escherichia coli	4JN4	Allosteric opening	X-RAY DIFFRACTION	37.03	10.1038/nsmb.2583
Escherichia coli	7KRU	Stimulating state of a truncated DnaK	X-RAY DIFFRACTION	34.35	10.1074/jbc.M708992200
Geobacillus kaustophilus	2V7Y	Post-ATP hydrolysis state	X-RAY DIFFRACTION	45.92	10.1074/jbc.M708992200
Homo sapiens	5E84	ATP-bound state	X-RAY DIFFRACTION	37.2	10.1016/j.str.2015.10.012
Homo sapiens	6ASY	ATP-bound state	X-RAY DIFFRACTION	36.32	10.1038/s41467-017-01310-z
Homo sapiens	7N1R	ATP hydrolysis to AMP (open conformation)	X-RAY DIFFRACTION	36.29	10.1002/pro.4267
Homo sapiens	3D2F	In complex with Sse1p	X-RAY DIFFRACTION	38.42	10.1016/j.cell.2008.05.022

Table S2. Phylogenetic distribution of 172 sequences.

Phylum	# bacteria
Acidobacteria	1
Actinobacterila	16
Aquificae	1
Bacteroidetes	11
Chlamydiae	2
Chlorobi	3
Chloroflexi	2
Cyanobacteria	7
Deinococcus-thermus	2
Dictyoglomi	1
Elusimicrobia	2
Firmicutes	**35**
Fusobacteria	1
Nitrospirae	1
Planctomycetes	1
Proteobacteria	**67**
Spirochaetes	3
Tenericutes	13
Thermotogae	1
Verrucomicrobia	2

Figure S4. First ML phylogenetic tree of 102 *Firmicutes* and *Proteobacteria* sequences with bootstrap values of nodes.

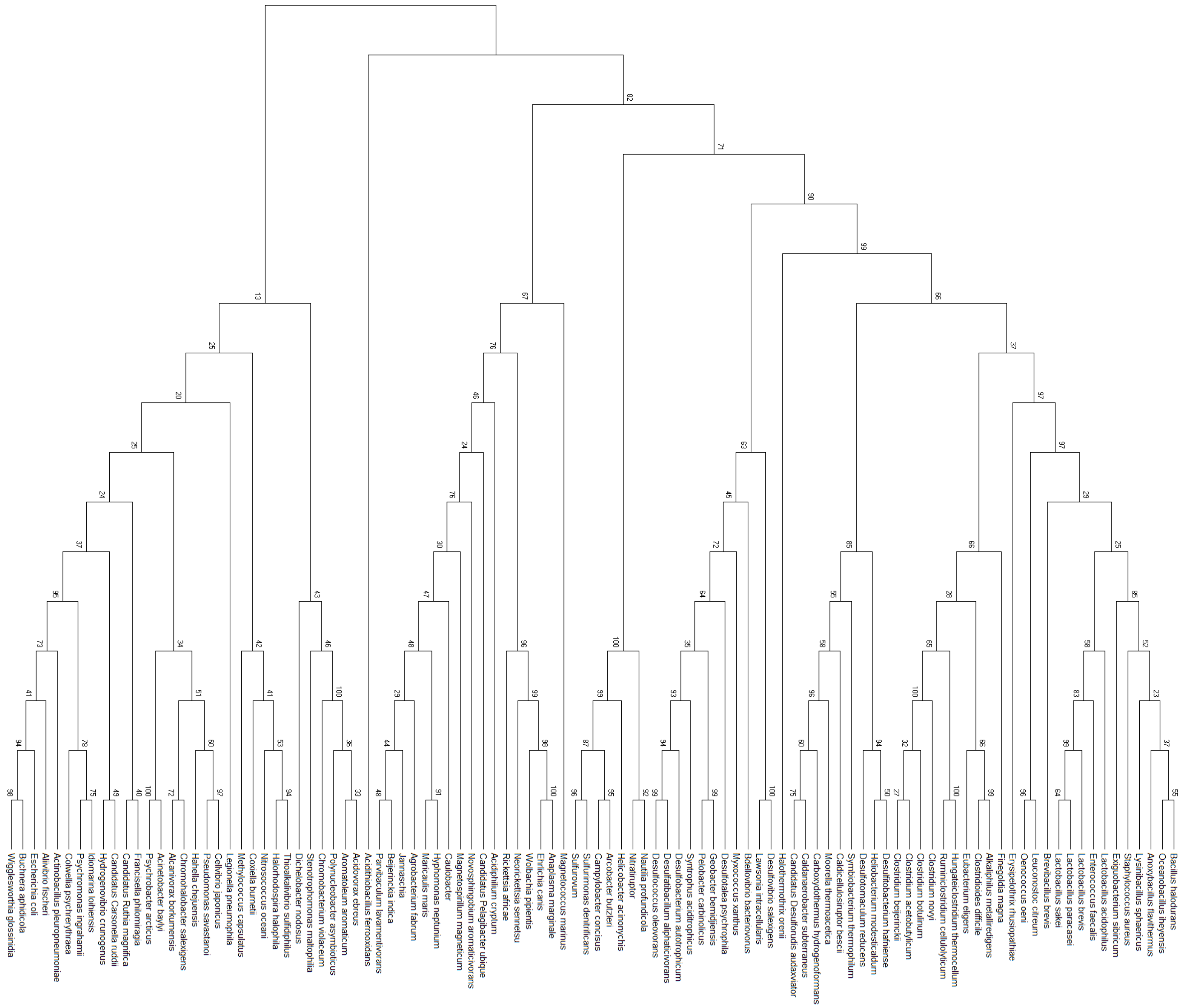

Figure S5. Condensed version of first ML phylogenetic tree of 102 *Firmicutes* and *Proteobacteria* sequences with bootstrap threshold set to 70%. 12 bacteria did not have any node (red color).

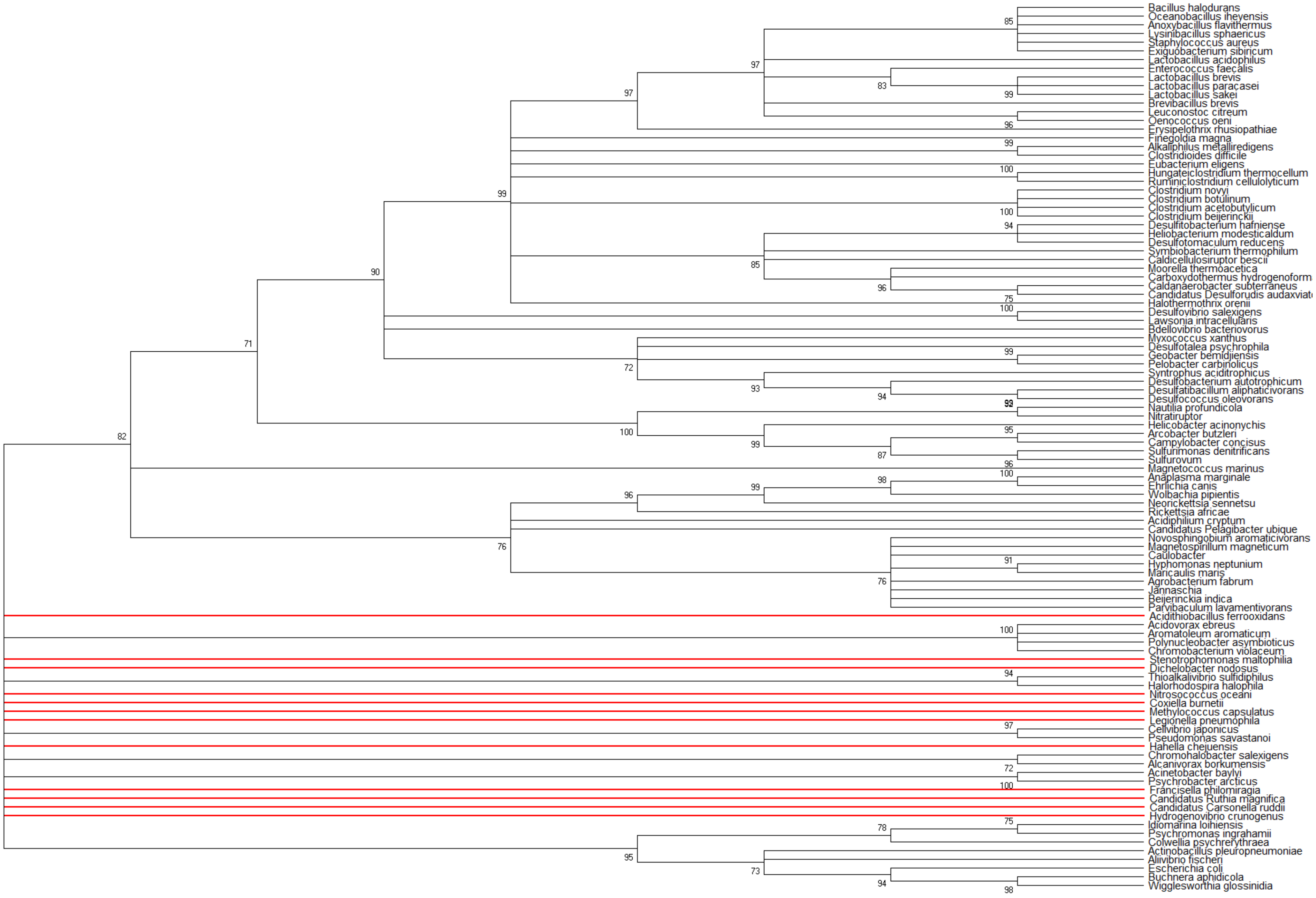

Table S3. Final list of 90 *Proteobacteria* and *Firmicutes* strains.

Proteobacteria			Firmicutes	
Buchnera aphidicola	*Aromatoleum aromaticum*	*Nitratiruptor*	*Halothermothrix orenii*	*Ruminiclostridium cellulolyticum*
Wigglesworthia glossinidia	*Magnetococcus marinus*	*Helicobacter acinonychis*	*Caldanaerobacter subterraneus*	*Erysipelothrix rhusiopathiae*
Escherichia coli	*Anaplasma marginale*	*Arcobacter butzleri*	*Carboxydothermus hydrogenoformans*	*Bacillus halodurans*
Actinobacillus pleuropneumoniae	*Ehrlichia canis*	*Campylobacter concisus*	*Candidatus Desulforudis audaxviator*	*Anoxybacillus flavithermus*
Aliivibrio fischeri	*Wolbachia pipientis*	*Sulfurimonas denitrificans*	*Moorella thermoacetica*	*Oceanobacillus iheyensis*
Colwellia psychrerythraea	*Neorickettsia sennetsu*	*Sulfurovum*	*Caldicellulosiruptor bescii*	*Staphylococcus aureus*
Idiomarina loihiensis	*Rickettsia africae*	*Desulfovibrio salexigens*	*Symbiobacterium thermophilum*	*Lysinibacillus sphaericus*
Psychromonas ingrahamii	*Candidatus Pelagibacter ubique*	*Lawsonia intracellularis*	*Desulfotomaculum reducens*	*Exiguobacterium sibiricum*
Acinetobacter baylyi	*Acidiphilium cryptum*	*Bdellovibrio bacteriovorus*	*Desulfitobacterium hafniense*	*Brevibacillus brevis*
Psychrobacter arcticus	*Novosphingobium aromaticivorans*	*Myxococcus xanthus*	*Heliobacterium modesticaldum*	*Leuconostoc citreum*
Chromohalobacter salexigens	*Caulobacter*	*Geobacter bemidjiensis*	*Clostridium acetobutylicum*	*Oenococcus oeni*
Alcanivorax borkumensis	*Magnetospirillum magneticum*	*Pelobacter carbinolicus*	*Clostridium beijerinckii*	*Lactobacillus acidophilus*
Cellvibrio japonicus	*Hyphomonas neptunium*	*Desulfotalea psychrophila*	*Clostridium botulinum*	*Enterococcus faecalis*
Pseudomonas savastanoi	*Maricaulis maris*	*Syntrophus aciditrophicus*	*Clostridium novyi*	*Lactobacillus brevis*
Thioalkalivibrio sulfidiphilus	*Jannaschia*	*Desulfobacterium autotrophicum*	*Finegoldia magna*	*Lactobacillus paracasei*
Halorhodospira halophila	*Parvibaculum lavamentivorans*	*Desulfatibacillum aliphaticivorans*	*Eubacterium eligens*	*Lactobacillus sakei*
Chromobacterium violaceum	*Agrobacterium fabrum*	*Desulfococcus oleovorans*	*Alkaliphilus metalliredigens*	
Polynucleobacter asymbioticus	*Beijerinckia indica*		*Clostridioides difficile*	
Acidovorax ebreus	*Nautilia profundicola*		*Hungateiclostridium thermocellum*	

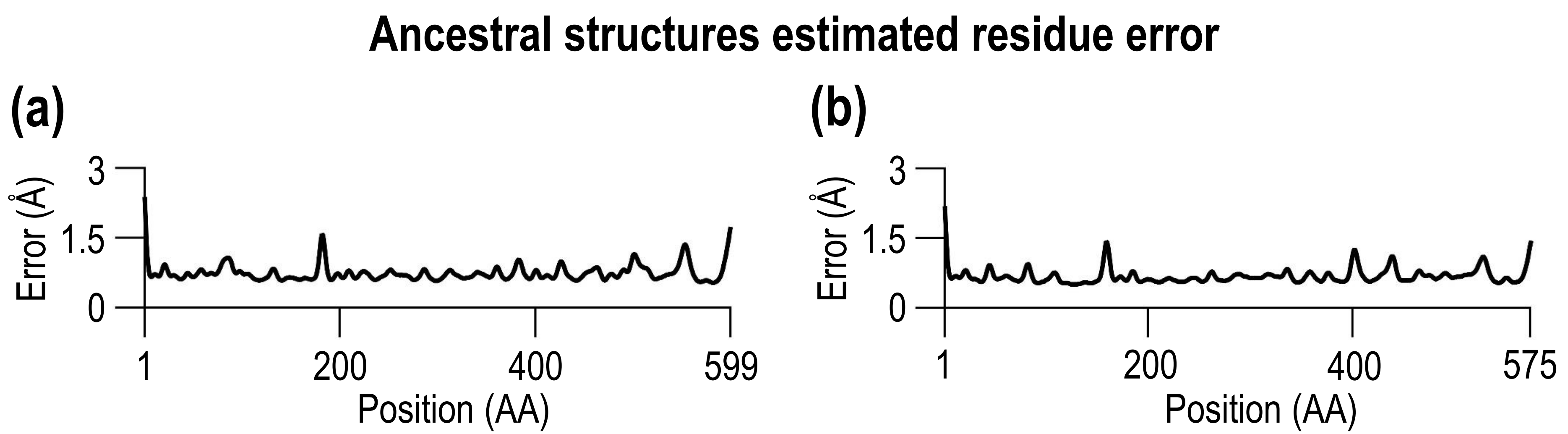

Figure S6. Ancestral structures estimated residue error. **(a)** Estimated error of *Proteobacteria* ancestral node 52. **(b)** Estimated error of *Firmicutes* ancestral node 45.

Table S4. List of calculation points for *Proteobacteria* divergence time estimation.

1. taxon	2. taxon	Min Time	Max Time	Estimated divergence time (MY)
Escherichia coli	*Buchnera aphidicola*	850	900	898.88
Colwellia psychrerythraea	*Psychromonas ingrahamii*	663.1	668.4	667.05
Buchnera aphidicola	*Psychromonas ingrahamii*	1673	2223	1697.25
Acinetobacter baylyi	*Pseudomonas savastanoi*	1306	1342	1326.21
Buchnera aphidicola	*Pseudomonas savastanoi*	1313	1753	1732.38
Chromobacterium violaceum	*Aromatoleum aromaticum*	1271.4	1834.4	1271.4
Buchnera aphidicola	*Aromatoleum aromaticum*	2138	2879	2138
Buchnera aphidicola	*Beijerinckia indica*	1707	2867	2676.9
Magnetospirillum magneticum	*Beijerinckia indica*	1886	2260	1886
Anaplasma marginale	*Beijerinckia indica*	1886	2260	2101.73
Buchnera aphidicola	*Sulfurovum*	2808	2981	2808
Helicobacter acinonychis	*Arcobacter butzleri*	841	1572	1001.18
Geobacter bemidjiensis	*Desulfococcus oleovorans*	2173	2420	2194.7
Desulfovibrio salexigens	*Desulfococcus oleovorans*	2309	2508	2508
Geobacter bemidjiensis	*Pelobacter carbinolicus*	1338.8	1412.9	1412.9
Myxococcus xanthus	*Geobacter bemidjiensis*	2173	2420	2237.41

Table S5. List of calculation points for *Firmicutes* divergence time estimation.

1. taxon	2. taxon	Min Time	Max Time	Estimated divergence time (MY)
Moorella thermoacetica	*Candidatus Desulforudis audaxviator*	1997	2381	1997
Desulfotomaculum reducens	*Desulfitobacterium hafniense*	1682	3181	2025.36
Clostridium novyi	*Clostridium botulinum*	196	2244	1134.1
Clostridioides difficile	*Alkaliphilus metalliredigens*	1823	3493	1823
Exiguobacterium sibiricum	*Bacillus halodurans*	1566	1814	1609.82
Lactobacillus sakei	*Halothermothrix orenii*	2482	3025	3025
Lactobacillus sakei	*Bacillus halodurans*	1698	2100	1777.1
Lactobacillus sakei	*Enterococcus faecalis*	805	1401	1243.95
Clostridioides difficile	*Eubacterium eligens*	1823	3493	2380.1

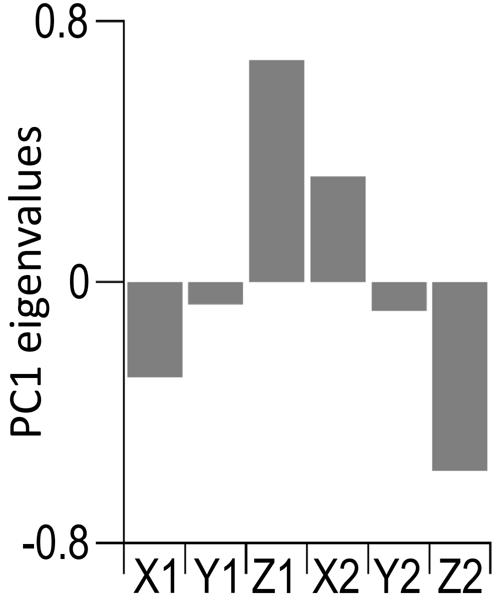

Figure S7. Eigenvalues of PC1. The highest space position variance for both contact residues show Z axis.
